# Effective-mononuclear cell (E-MNC) therapy alleviates salivary gland damage by suppressing lymphocyte infiltration in Sjögren-like disease

**DOI:** 10.3389/fbioe.2023.1144624

**Published:** 2023-04-24

**Authors:** Kayo Hasegawa, Jorge Luis Montenegro Raudales, Takashi I, Takako Yoshida, Ryo Honma, Mayumi Iwatake, Simon D. Tran, Makoto Seki, Izumi Asahina, Yoshinori Sumita

**Affiliations:** ^1^ Department of Medical Research and Development for Oral Disease, Nagasaki University Graduate School of Biomedical Sciences, Nagasaki, Japan; ^2^ Unit of Translational Medicine, Department of Regenerative Oral Surgery, Nagasaki University Graduate School of Biomedical Sciences, Nagasaki, Japan; ^3^ Laboratory of Craniofacial Tissue Engineering and Stem Cells, Faculty of Dental Medicine and Oral Health Sciences, McGill University, Montreal, QC, Canada; ^4^ CellAxia Inc, Tokyo, Japan; ^5^ Depatment of Oral and Maxillofacial Surgery, Juntendo University Hospital, Tokyo, Japan

**Keywords:** Sjögren syndrome, xerostomia, cell therapy, peripheral blood mononuclear cell, macrophage

## Abstract

**Introduction:** Sjögren syndrome (SS) is an autoimmune disease characterized by salivary gland (SG) destruction leading to loss of secretory function. A hallmark of the disease is the presence of focal lymphocyte infiltration in SGs, which is predominantly composed of T cells. Currently, there are no effective therapies for SS. Recently, we demonstrated that a newly developed therapy using effective-mononuclear cells (E-MNCs) improved the function of radiation-injured SGs due to anti-inflammatory and regenerative effects. In this study, we investigated whether E-MNCs could ameliorate disease development in non-obese diabetic (NOD) mice as a model for primary SS.

**Methods:** E-MNCs were obtained from peripheral blood mononuclear cells (PBMNCs) cultured for 7 days in serum-free medium supplemented with five specific recombinant proteins (5G culture). The anti-inflammatory characteristics of E-MNCs were then analyzed using a co-culture system with CD3/CD28-stimulated PBMNCs. To evaluate the therapeutic efficacy of E-MNCs against SS onset, E-MNCs were transplanted into SGs of NOD mice. Subsequently, saliva secretion, histological, and gene expression analyses of harvested SG were performed to investigate if E-MNCs therapy delays disease development.

**Results:** First, we characterized that both human and mouse E-MNCs exhibited induction of CD11b/CD206-positive cells (M2 macrophages) and that human E-MNCs could inhibit inflammatory gene expressions in CD3/CD28- stimulated PBMNCs. Further analyses revealed that Msr1-and galectin3-positive macrophages (immunomodulatory M2c phenotype) were specifically induced in E-MNCs of both NOD and MHC class I-matched mice. Transplanted E-MNCs induced M2 macrophages and reduced the expression of T cell-derived chemokine-related and inflammatory genes in SG tissue of NOD mice at SS-onset. Then, E-MNCs suppressed the infiltration of CD4-positive T cells and facilitated the maintenance of saliva secretion for up to 12 weeks after E-MNC administration.

**Discussion:** Thus, the immunomodulatory actions of E-MNCs could be part of a therapeutic strategy targeting the early stage of primary SS.

## 1 Introduction

Primary Sjögren syndrome (pSS) is a systemic autoimmune disease characterized by lymphocytic infiltration of the exocrine glands, primarily the salivary and lacrimal glands, leading to xerostomia and eye dryness (Ramos-Casals et al., 2012; Nocturne and Mariette, 2013). In addition, there are many systemic features of pSS, such as Raynaud’s syndrome, fatigue, dry skin, and joint and muscular pain, and these symptoms seriously affect patients’ quality of life (Brito-Zerón et al., 2016; Both et al., 2017). Therefore, scientists have sought to develop effective treatments, and during this process, much progress has been made in disclosing the pathogenesis of SS. However, the etiology of SS remains unclear owing to its complexity. Consequently, there are currently no adequate treatments that do not involve suppression of autoimmune responses, and therefore, developing an adequate treatment for SS is urgently needed.

Over the past decade, cell-based therapies have been examined as potential therapeutic approaches for SS (Khalili et al., 2014; Elghanam et al., 2017; Genç et al., 2022). A particularly promising therapeutic option is the use of mesenchymal stem cells (MSCs). MSCs originating from either the bone marrow, umbilical cord, or adipose tissues have shown potential in the treatment of SS due to their anti-inflammatory and immunomodulatory effects (Khalili et al., 2012; Yao et al., 2019; Liu et al., 2021). For example, we have shown that a cell-based therapy using bone marrow cells or bone marrow MSCs (BMMSCs) could prevent SS in a non-obese diabetic (NOD) mouse model (Khalili et al., 2012; Khalili et al., 2014). BMMSCs (CD45^−^/TER119^−^) prevented the loss of saliva flow and reduced lymphocytic infiltrations in salivary glands (SGs). In the developing stages of SS, the production of inflammatory cytokines such as tumor necrosis factor (TNF)-α and transforming growth factor (TGF)-β is usually elevated, but expression of these cytokines was downregulated in NOD mice treated with BMMSCs (Jonsson et al., 2006; Hall et al., 2010). MSCs reportedly secrete anti-inflammatory vesicles and suppress the immune system (Chang et al., 2021). These findings demonstrated that bone marrow cells or BMMSCs reduce lymphocytic infiltration and improve the saliva secretory function in mice with Sjögren-like disease (Khalili et al., 2012; Khalili et al., 2014). However, the clinical use of BMMSCs carries some risks. For example, although these cells possess attractive self-renewal and vigorous proliferation capacities, these properties may also lead to undesirable consequences such as tumor formation or progression (Norozi et al., 2016; Clément et al., 2017). Therefore, we subsequently examined the therapeutic potential of bone marrow cell- or MSC-extract/lysate (soup) on atrophic irradiated SGs or SGs from mice with SS-like disease (Misuno et al., 2014; Abughanam et al., 2019). An extract of the soluble intracellular contents of bone marrow cells or MSCs exhibited immunomodulatory effects in NOD mice by inhibiting the production of pro-inflammatory cytokines such as TNF-α, TGF-β1, and interleukin (IL)-1β and promoting the secretion of IL-10. However, it remains a challenge to expand a sufficient number of highly-functional MSCs in culture to obtain an adequate amount of cell extract/lysate from autologous MSCs. This is because there are large individual differences in cell proliferation and function caused by the patient’s age, gender, and/or underlying medical conditions (Agata et al., 2010; 2019; Asahina et al., 2021). In addition, various unavoidable problems such as cytotoxicity against host immune cells or increased tumor recurrence associated with immunosuppressive effects remain with the use of allogenic MSCs (Norozi et al., 2016; Clément et al., 2017). Further investigation is thus required to develop a highly effective cell-based therapy for SS patients.

We recently developed an effective culture method that enhances the anti-inflammatory and vasculogenic phenotypes of peripheral blood mononuclear cells (PBMNCs) (Kuroshima et al., 2019; I et al., 2019). Mouse PBMNCs were expanded for 5 days in primary serum-free culture medium containing five specific recombinant proteins (which we designated as “5G-culture”), and then the resulting effectively conditioned mononuclear cells [effective-mononuclear cells (E-MNCs)] could be obtained. E-MNCs contained an enriched population of CD11b/CD206-positive (M2 macrophage-like) cells and CCR4^+^/CCR6^−^ cells (Th2 cells), and this characteristic change strongly suggested that PBMNCs acquire anti-inflammatory or immunomodulatory properties during 5G-culture. Indeed, experiments to confirm the efficacy of E-MNC transplantation on radiation-damaged SGs showed that it reduced the expression of inflammation-related genes and promoted various tissue-regenerative activities, such as increased expression of stem cell markers, activation of cell proliferation, and vigorous blood vessel formation. Subsequently, through the induction of acinar and ductal cell proliferation and the suppression of fibrosis, E-MNCs ultimately mediated the regeneration of damaged tissues in mice, leading to the recovery of saliva secretory function (I et al., 2019). Thus, E-MNC transplantation is a promising approach for the treatment of radiation-injured SGs, and a first-in-humans study of E-MNC therapy for patients with severe radiogenic xerostomia is currently in progress (Sumita et al., 2020). Moreover, several studies have reported that treatment with M2-macrophages exhibiting anti-inflammatory and immunomodulatory properties can alleviate the symptoms of autoimmune diseases such as glomerulonephritis, multiple sclerosis, and encephalomyelitis (Du et al., 2016; Chu et al., 2021). Therefore, we hypothesized that E-MNCs would be therapeutically efficient against autoimmune diseases such as SS.

The aim of this study was to investigate whether E-MNCs could hinder the development of SS-like disease and preserve the saliva secretory function in NOD mice. This study is a pre-requisite for future clinical trials aimed at developing cell-based therapies for SS.

## 2 Materials and methods

### 2.1 Animals

CB6F1/Slc mice (Japan SLC Inc., Shizuoka, Japan) and NOD/ShiJcl mice (CLEA Japan Inc., Tokyo, Japan) were used for E-MNC culture. In transplantation experiments, female NOD/ShiJcl mice with Sjögren-like disease were used as recipients (CLEA Japan Inc.), and male CB6F1/Slc mice in which the MHC class I matched that of NOD mice were employed as donors. Starting at 8 weeks of age, body weight and blood glucose levels of NOD mice were monitored once a week (Accu-Check; Roche Diagnostics, Laval, QC, Canada). The mice were diagnosed with diabetes after observing two consecutive daily blood glucose concentration measurements of >200 mg/dL. These diabetic mice were injected with insulin on a daily basis (Humulin N, Lilly, ON, Canada). All mice were kept under clean conventional conditions at the Nagasaki University Animal Center. All experimental procedures were performed in accordance with the guidelines approved by the Nagasaki University Ethics Committee (1605061303, 1812141494, and 1912111584).

### 2.2 E-MNC culture

Human PBMNCs were cultured using a specific culture method (CellAxia Inc. Tokyo, Japan) that we established and designated as “5G-culture” (I et al., 2019). Briefly, 50 mL of peripheral blood was obtained from healthy volunteers, and PBMNCs were isolated by density gradient centrifugation using Histopaque^®^-1,077 separating solution (Sigma Aldrich, St. Louis, MO, United States). PBMNCs were seeded into 6-well Primaria tissue culture plates (BD Biosciences, San Jose, CA, United States) at a density of 2 × 10^6^ cells/well in 2 mL of serum-free medium (Stemline II Hematopoietic Stem Cell Expansion Medium; Sigma Aldrich) supplemented with five recombinant proteins [Flt3-ligand, IL-6, stem cell factor (SCF), thrombopoietin (TPO), and vascular endothelial growth factor (VEGF)] and cultured for 6 days. Experiments were carried out in compliance with the Declaration of Helsinki. Sample collection was approved by the Ethics Committee of Nagasaki University Graduate School of Biomedical Sciences (17082131), and written informed consent was obtained from all donors. For mouse E-MNC culture, PBMNCs were isolated from heparinized blood samples obtained by cardiac puncture and density gradient centrifugation with Histopaque^®^-1,083 (Sigma Aldrich). PBMNCs were cultured for 7 days as described above with equivalent mouse recombinant proteins at a seeding density of 5 × 10^6^ cells/well. The concentrations of human and mouse recombinant proteins used in the 5G medium are listed in Table 1.

**TABLE 1:** Growth factors used in 5G culture.

Antibody/isotype name	Company, catalog no.	Final concentration (ng/mL)
h SCF	Prepotech, # AF-300-07	100
h flt-3 ligand	Prepotech, #AF- 300-19	100
h TPO	Prepotech, #AF-300-18	20
h VEFG	Prepotech, # AF-100-20	50
h IL-6	Prepotech, # AF-200-06	20
m SCF	Prepotech, #250-03	100
m flt-3 ligand	Prepotech, #250-31L	100
m TPO	Prepotech, #315-14	20
m VEFG	Prepotech, #450-32	50
m IL-6	Prepotech, #216-16	20

h: human, m: mouse.

### 2.3 Evaluation of the characteristics of E-MNCs

Freshly isolated PBMNCs and E-MNCs (human and mouse) were subjected to flow cytometry to determine the surface antigen positivity of macrophage subpopulations (human monocytes and/or naïve macrophages: CD11b^+^/CD206^−^; human M2 macrophages: CD11b^+^/CD206^+^) (mouse monocytes and/or naïve macrophages: CD11b^+^/CD206^−^; mouse M2 macrophages: CD11b^+^/CD206^+^, CD11b^+^/macrophage scavenger receptor 1 (Msr1)^+^ or CD11b^+^/CCR2^−^/galectin3^+^) (Shirakawa et al., 2018) and T lymphocyte (CD3^+^/CD4^+^) subsets (mouse Th1 cells: CXCR3^+^; mouse Th2 cells: CCR4^+^/CCR6^−^). The relevant antibodies are listed in Table 2. Cells were resuspended in 2 mmol/L of EDTA/0.2% BSA/PBS buffer (2 × 10^5^ cells/200 μL). Flow cytometry analysis was performed using an LSRFortessa cell analyzer (BD Biosciences) and FlowJo software (Tomy Digital Biology Co., Ltd., Tokyo, Japan). The percent positivity of macrophage and T lymphocytes subpopulations per each gate among PBMNCs or E-MNCs was evaluated and then calculated in relation to that of the whole cell population.

**TABLE 2:** Antibodies and isotype controls for flow cytometry.

Antibody/isotype name	Company, catalog no.
PE-Cy7 anti-human CD11b	Biolegend, # 301322
PE-Cy7 mouse IgG1, κ isotype Ctrl	Biolegend, # 400126
APC-Cy7 anti-human CD206	Biolegend, # 321120
APC-Cy7 mouse IgG1, κ Isotype Ctrl	Biolegend, # 400128
APC-Cy7 anti-mouse/human CD11b	Biolegend, # 101225
APC-Cy7 rat IgG2b, κ Isotype Ctrl	Biolegend, # 400623
APC anti-mouse CD206 (MMR)	Biolegend, # 141707
APC rat IgG2a, κ isotype Ctrl	Biolegend, # 400713
PE anti-mouse CD3	Biolegend, # 100205
PE Rat IgG2b, κ Isotype Ctrl	Biolegend, # 400607
APC-Cy7 anti-mouse CD4	Biolegend, # 100413
APC-Cy7 Rat IgG2b, κ Isotype Ctrl	Biolegend, # 400623
PE-Cy7 anti-mouse CCR4 (CD194)	Biolegend, # 131213
PE-Cy7 Armenian hamster IgG, isotype Ctrl	Biolegend, # 400921
APC anti-mouse CCR6 (CD196)	Biolegend, # 129813
APC Armenian hamster IgG, isotype Ctrl	Biolegend, # 400911
APC anti-mouse CCR2 (CD192)	R&D Systems, #FAB5538A
APC Rat IgG2b, κ Isotype Ctrl	Biolegend, # 400611
PE-Cy7 anti-mouse/human Mac-2 (Galectin-3)	Biolegend, # 125417
PE-Cy7 rat IgG2a, κ isotype Ctrl	Biolegend, # 400521
FITC anti-mouse Msr-1 (CD204)	Bio-rad, # MCA1322FT
FITC rat IgG2b, κ isotype Ctrl	BD biosciences, # 553988

### 2.4 Evaluation of anti-inflammatory potential of E-MNCs

To assess the anti-inflammatory potential of E-MNCs, an assay using co-culture conditions with T lymphocyte–stimulated PBMNCs was utilized. The wells of a 24-well plate were coated overnight with 15 ng/mL of CD3 monoclonal antibody (eBioscience, Vienna, Austria) and 5 ng/mL of CD28 monoclonal antibody (eBioscience). Human PBMNCs were isolated from healthy donors (three men aged 28, 34, and 35 years) as described above and seeded in the pre-coated 24-well plates (2.5 × 10^6^ cells/well) in 1 mL of RPMI 1640 medium (Thermo Fisher Scientific Life Sciences, Waltham, MA, United States). After 1 h of incubation at 37°C, a Transwell^®^ 0.4-µm pore polycarbonate membrane insert (Corning Life Sciences, Amsterdam, Netherlands) was placed above the seeded PBMNCs. E-MNCs (5 × 10^6^ cells) were added to the inside compartment of the Transwell^®^ and co-cultured without cell-to-cell contact between E-MNCs and PBMNCs for 1 and 3 h. Finally, PBMNCs were collected, and total RNA was extracted using Trizol reagent (Invitrogen) to assess mRNA expression of the *tnf-α*, *interferon (inf)-γ*, *il-1β*, *il-6*, *il-4*, and *il-10* genes.

### 2.5 Evaluation of vasculogenic potential of E-MNCs, and gene expression of cells and tissues

To investigate the vasculogenic potential of PBMNCs (pre–5G-culture) obtained from CB6F1/Slc mice and E-MNCs, the cells were seeded in 35-mm Primaria dishes (BD Biosciences) at 1 × 10^5^ cells/dish. Endothelial progenitor cell–colony forming assays (EPC-CFAs) were performed using semi-solid culture medium (MethoCult SFBIT; STEMCELL Technologies, Inc., Vancouver, Canada) with pro-angiogenic growth factors/cytokines (Table 3), as we previously reported (Masuda et al., 2011; I et al., 2019). Briefly, 7 days after initiation of the EPC-CFA, the number of adherent colonies (EPC colony-forming units; EPC-CFUs) per dish was determined under a microscope. The EPC-CFA was adopted to monitor two different types of EPC-CFUs, as primitive EPC-CFUs (PEPC-CFUs) and definitive EPC-CFUs (DEPC-CFUs), which were composed of small cells and large cells, respectively. The PEPC-CFUs and DEPC-CFUs were counted separately. These analyses were repeated six times for PBMNCs and E-MNCs. Simultaneously, to confirm the endothelial characteristics of colonized cells, we used fluorescence microscopy to assess the biochemical binding of isolectin B4–conjugated fluorescein isothiocyanate (ILB4-FITC; Vector Laboratories, Burlingame, CA, United States), which is a marker of endothelial cells, and the uptake of acetylated low-density lipoprotein labeled with 1,1′-dioctadecyl-3,3,3′,3′-tetramethylindo- carbocyanine perchlorate (AcLDL-DiI; Biomedical Technologies, Inc., Stoughton, MA, United States), which is metabolized in endothelial cells via a receptor-mediated process.

**TABLE 3:** Contents of semisolid culture medium used for EPC-CFA.

Contents	Company, catalog no.	Final concentration
VEGF	Peprotech, #450-32	50 ng/mL
basic FGF	Peprotech, #450-33	50 ng/mL
EGF	Peprotech, #315-09	50 ng/mL
IGF-1	Peprotech, #250-19	50 ng/mL
SCF	Peprotech, #250-03	100 ng/mL
IL-3	Peprotech, #213-13	20 ng/mL
Heparin	YOSHINDO, #121-154	2 U/mL
FBS	SAFC Biosciences, #12303	30%

To analyze the gene expression in PBMNCs, E-MNCs, and submandibular glands, total RNA was extracted using Trizol reagent (Invitrogen, Waltham, MA, United States), and first-strand complementary DNA synthesis was performed using SuperScript First-Strand Synthesis (Invitrogen). Complementary DNA was amplified using Takara-Taq (Takara Bio Inc., Shiga, Japan). PCR reactions were performed on an Mx3000P QPCR System (Agilent Technologies). The primers used for the reactions are shown in Table 4. As an internal control standard, glyceraldehyde-3-phosphate dehydrogenase (gapdh) was used in both human and mouse reactions.

**TABLE 4:** Primer sequences used for quantitative PCR analysis.

Gene	Forward primer	Reverse primer
Human		
*gapdh*	5′-GGA​GTC​CAC​TGG​CGT​CTT​CAC-3′	5′-GCT​GAT​GAT​CTT​GAG​GCT​GTT​GTC-3′
*tnf-α*	5′-AAT​GGC​GTG​GAG​CTG​AGA-3′	5′-TAG​ACC​TGC​CCA​GAC​TCG​G-3′
*inf-γ*	5′-CTG​TTA​CTG​CCA​GGA​CCC​AT-3′	5′-ACA​CTC​TTT​TGG​ATG​CTC​TGG​T-3′
*il-1β*	5′-CAC​AGA​CCT​TCC​AGG​AGA​AT-3′	5′-TTC​AAC​ACG​CAG​GAC​AGG​TA-3′
*il-6*	5′-GTA​CAT​CCT​CGA​CGG​CAT​C-3′	5′-AGC​CAC​TGG​TTC​TGT​GCC​T-3′
*il-4*	5′-GCC​ACC​ATG​AGA​AGG​ACA​CT-3′	5′-ACT​CTG​GTT​GGC​TTC​CTT​CA-3
*il-10*	5′-TGA​AAA​CAA​AGA​GCA​AGG​CCG-3′	5′-TAG​AGT​CGC​CAC​CCT​GAT​GT-3
Mouse		
*gapdh*	5′-TGT​GTC​CGT​CGT​GGA​TCT​GA-3′	5′-TTG​CTG​TTG​AAG​TCG​CAG​GAG-3′
*tnf-α*	5′-CCA​CCA​CGC​TCT​TCT​GTC​TA-3′	5′-AGG​GTC​TGG​GCC​ATA​GAA​CT-3′
*inf-γ*	5′-ACA​GCA​AGG​CGA​AAA​AGG​ATG-3′	5′-TGG​TGG​ACC​ACT​CGG​ATG​CA-3′
*il-1β*	5′-GCT​GAA​AGC​TCT​CCA​CCT​CA-3′	5′-AGG​CCA​CAG​GTA​TTT​TGT​CG-3′
*ccl5*	5′-TGC​TGC​TTT​GCC​TAC​CTC​TC-3′	5′-TCC​TTC​GAG​TGA​CAA​ACA​CGA-3′
*ccl6*	5′-CAA​GCC​GGG​CAT​CAT​CTT​TA-3′	5′-TTC​CCA​GAT​CTT​GGG​CCT​TG-3′
*ccl8*	5′-ACG​CTA​GCC​TTC​ACT​CCA​AA-3′	5′-GTG​ACT​GGA​GCC​TTA​TCT​GG-3′
*ccl19*	5′-AGA​CTG​CTG​CCT​GTC​TGT​GA-3′	5′-GCC​TTT​GTT​CTT​GGC​AGA​AG-3′
*cxcl19*	5′-CTC​GGA​CTT​CAC​TCC​AAC​ACA-3′	5′-ATC​ACT​AGG​GTT​CCT​CGA​ACT-3′

### 2.6 Microarray analysis of PBMNCs and E-MNCs, and transplantation of E-MNCs

For microarray analysis, PBMNCs and E-MNCs obtained from CB6F1 mice were collected, and total RNA was extracted. Each RNA sample was assessed using a microarray (SurePrint G3 Human Gene Expression 8 × 60 K v2.0; Agilent Technologies, Palo Alto, CA, United States). Differentially expressed genes were selected and divided into functional categories using Database for Annotation, Visualization, and Integrated Discovery (DAVID) (https://david.ncifcrf.gov/). The signal intensity of highly expressed genes was converted into z scores and plotted in a heatmap using GraphPad Prism, version 9.4.1 (GraphPad Software; San Diego, CA, United States).

For transplantation of E-MNCs, eight-week-old female NOD mice were anesthetized with 0.1 mL/10 g body weight of mixed anesthetic agents (domitor, 1 mg/mL; midazolam, 10 mg/2 mL; and butorphanol tartrate, 5 mg/mL) given by intraperitoneal (ip) injection. E-MNCs (1 × 10^5^ cells) obtained from male CB6F1/Slc mice were suspended in 5 µL of IMDM medium (Sigma Aldrich) and injected into the submandibular glands of NOD mice (E-MNC-group; sacrificed at 1, 3, and 7 days, and 4, 8, and 12 weeks post-transplantation, *n* ≥ 5 for each time point, total *n* ≥ 30). In control mice, an equal volume of IMDM without cells was injected into the SGs (Ctrl-group; n ≥ 5 for each time point, sacrificed at 1, 3, and 7 days, and 4, 8, and 12 weeks post-transplantation, *n* ≥ 5 for each time point, total *n* ≥ 30). At 4, 8, and 12 weeks post-transplantation, mice were sacrificed after collecting saliva, and the submandibular glands were harvested for histological observations. To track transplanted E-MNCs, the cells were labeled using a PKH26 Red Fluorescent Cell Linker kit (Sigma Aldrich) prior to transplantation. The same mice then received transplantation of E-MNCs in one gland (right side) and only IMDM, as a control, in the other gland (left side) for further gene expression (*ccl6*, *ccl8*, *ccl5*, *ccl19*, *ccl9*, *tnf-α*, *inf-γ*, and *il-1β*) and histological (CD206) analyses. Subsequently, the submandibular glands were harvested at 1, 3, and 7 days after transplantation (*n* = 3 at each time point).

### 2.7 Salivary flow rate (SFR) after transplantation

To measure the saliva secretory function (SFR) of SGs, mice were kept under general anesthesia via ip injection of 10 μL/g body weight of triple anesthetic combination. Whole saliva was collected after stimulation of secretion with 0.5 mg/kg body weight pilocarpine (Sigma Aldrich) administered subcutaneously, as previously described (Sumita et al., 2010; I et al., 2019). Saliva was obtained from the oral cavity using a micropipette and placed into pre-weighed 1.5-mL microcentrifuge tubes. Saliva was collected for a 10-min period, and the volume was determined gravimetrically. SFR was determined at 4, 8, and 12 weeks post-transplantation (*n* = 8/group at each time point).

### 2.8 Epidermal growth factor (EGF) concentration

The concentration of EGF, which stimulates the proliferation of epithelial cells, in saliva (n = 3/group in Ctrl- and E-MNC–group) was measured by ELISA (Abcam). This assay employed a quantitative sandwich enzyme immunoassay technique, with the intensity of the color measured being proportional to the amount of EGF. The sample values were compared to an EGF standard curve. EGF concentration was determined at 12 weeks post-transplantation.

### 2.9 Histological observations of submandibular glands

Harvested submandibular glands were fixed in 4% paraformaldehyde and embedded in paraffin. Sections (5 µm) were stained with hematoxylin and eosin (H&E) and examined microscopically under ×100 magnification, with analysis of five different specimens per mouse (*n* = 5/group at 4, 8, and 12 weeks post-transplantation). The percentage of lymphocyte infiltrate area (focus score area) was analyzed using ImageJ software (National Institutes of Health, Bethesda, MD, United States). Immunohistological staining was performed using rat anti-mouse F4/80 antibody (1:100, Bio-Rad Laboratories, Inc., Hercules, CA, United States), rabbit anti-mouse CD206 antibody (1:500, Abcam, Cambridge, United Kingdom), and rat anti-mouse CD4 antibody (1:50; R&D Systems, Minneapolis, MN, United States). The slides were then incubated with Alexa Fluor 647–conjugated goat anti-rat antibody (1:200; Invitrogen™, Carlsbad, CA, United States) for F4/80 and Alexa Fluor 488–conjugated donkey anti-rabbit antibody (1:200; Invitrogen™) for CD206 as secondary antibodies and counterstained with mounting medium for fluorescence with DAPI (Vector Laboratories). Specimens stained with F4/80 and CD206 antibodies were examined microscopically under ×40, ×200, and ×400 magnification, with five different specimens per mouse (*n* = 3/group at 1, 3, and 7 days post-transplantation). Two examiners independently counted CD206-positive cells in a blinded manner. For CD4 staining, a VECTASTAIN ABC kit (Vector laboratories) was used, and specimens were counterstained with hematoxylin. CD4-positive cells were examined using a fluorescence microscope under ×100, ×200, and ×400 magnification. Two examiners independently counted CD4-positive cells in the lymphocytic infiltrate of each specimen/four sections (*n* = 3/group at 4 and 8 weeks after transplantation) in a blinded manner.

### 2.10 Statistical analysis

The Student’s t-test was used to determine the significance of differences between paired data, and one-way ANOVA with *post hoc* Tukey’s multiple comparisons were performed for multiple groups. Experimental values are presented as mean ± SD; *p* < 0.05 was considered statistically significant.

## 3 Results

### 3.1 Characteristics of human E-MNCs

**FIGURE 1:**
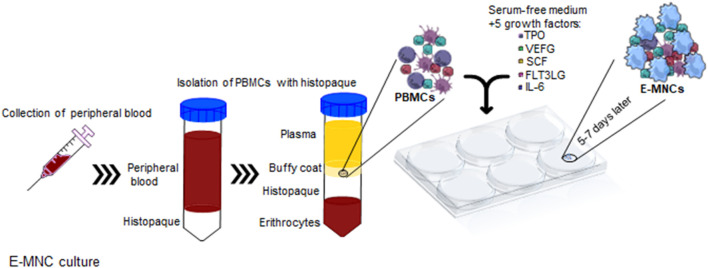
Schematic diagram describing 5G-culture. PBMNCs were isolated from buffy coat and cultured for 6–7 days in serum-free medium supplemented with five recombinant proteins, TPO, VEGF, SCF, Flt-3 ligand, and IL-6. After cultivation, effective-mononuclear cells (E-MNCs) were obtained.

**FIGURE 2:**
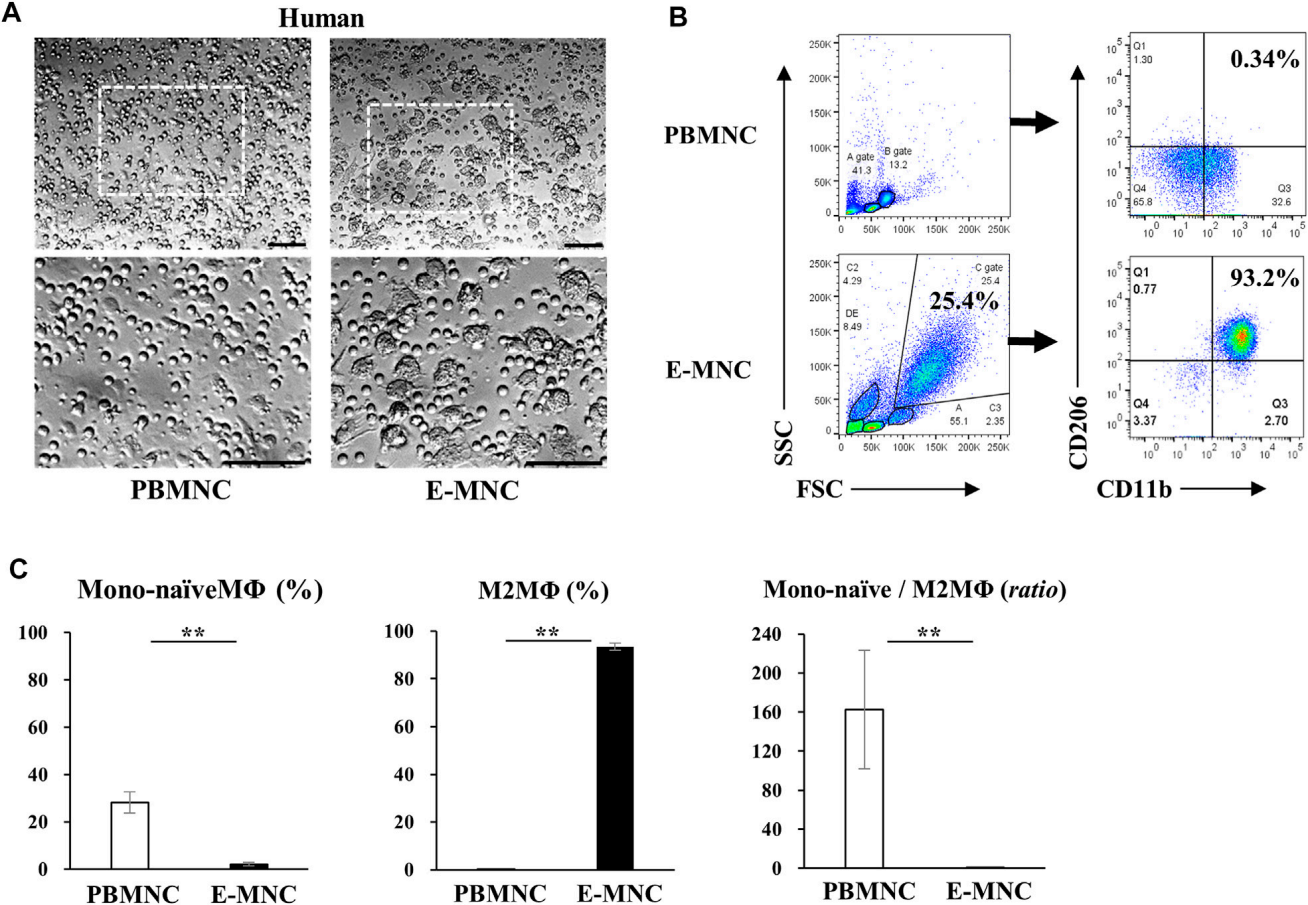
Characteristics of human E-MNCs. **(A)** Phase-contrast imaging of human PBMNCs (at day 0) and E-MNCs (at day 6). White boxed areas in the upper images (scale bar, 100 µm) were magnified in the lower images (scale bar, 100 µm). **(B)** Flow cytometric analysis of FSC/SSC gated cells, CD11b^+^/CD206^−^ [Monocytes and/or naïve (Mono-naïve) macrophages] and CD11b^+^/CD206^+^ (M2) macrophages, among PBMNCs (at day 0) and E-MNCs (at day 6). **(C)** Percentages of Mono-naïve (CD11b^+^/CD206^−^) and M2 (CD11b^+^/CD206^+^) macrophage fractions and their ratios (Mono-naïve/M2) among PBMNCs and E-MNCs (**p < 0.01). For statistical analysis, the Student’s t-test was performed to determine the significance of differences among PBMNCs and E-MNCs.

At 6 days of 5G-culture (Figure 1), adherent human E-MNCs became larger and changed their morphology to a macrophage-like round shape (Figure 2A). Flow cytometric analysis showed a distinct increase in FSC/SSC (cell size/granularity) gated cells, and the M2 macrophage–enriched fraction (CD11b^+^/CD206^+^) distinctly appeared among E-MNCs (from 0.20% ± 0.12% to 93.47% ± 1.62%) (Figures 2B, C). In contrast, monocytes and/or naïve (Mono-naïve) macrophages (CD11b^+^/CD206^−^) decreased (from 28.2% ± 4.56% to 2.09% ± 0.74%), and the Mono-naïve/M2 macrophage ratio in E-MNCs decreased markedly compared with that in PBMNCs (from 162.55 ± 60.4 to 0.02 ± 0.01) (Figure 2C).

### 3.2 Characteristics of mouse E-MNCs

**FIGURE 3:**
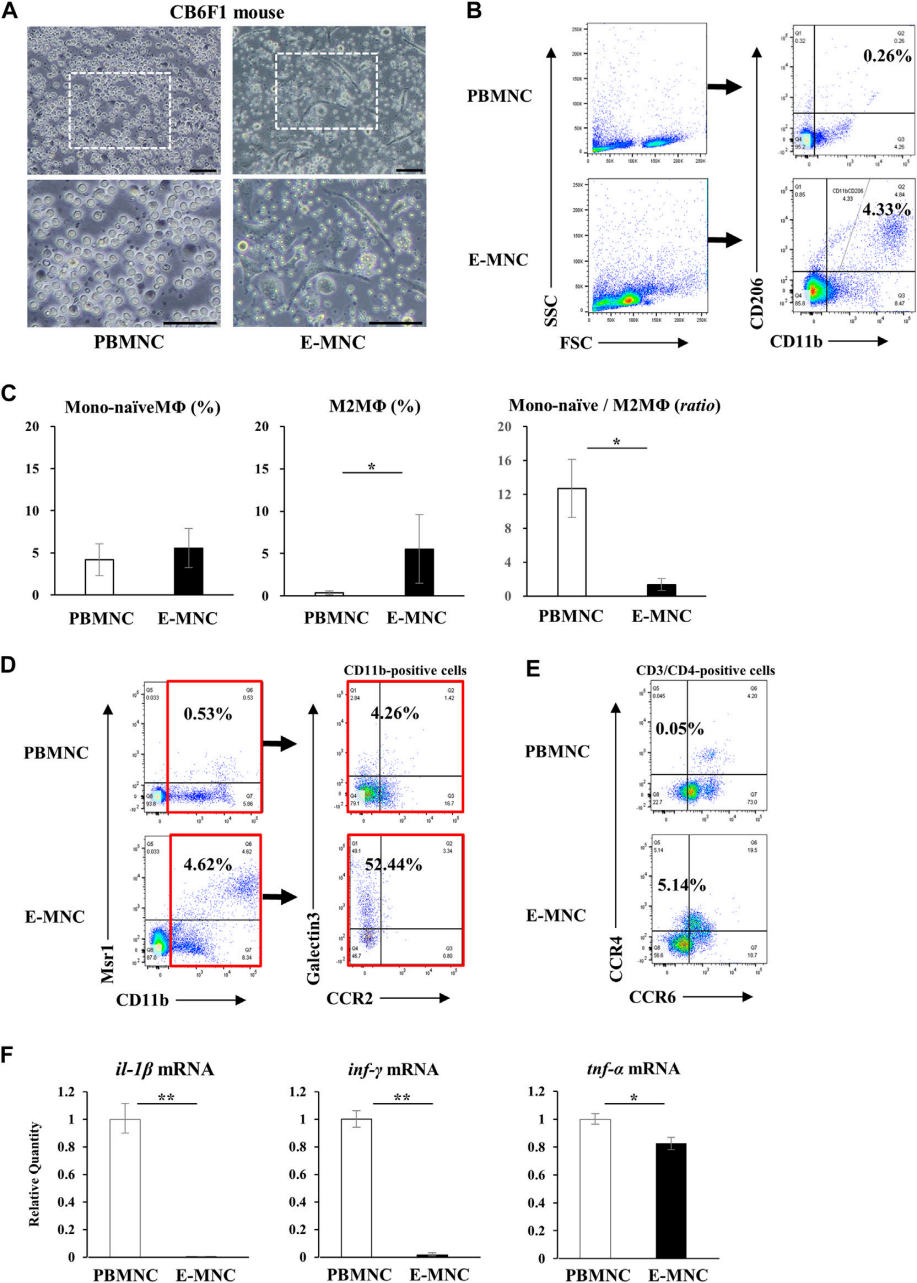
Characteristics of mouse E-MNCs. **(A)** Phase-contrast imaging of CB6F1 mouse PBMNCs (at day 0) and E-MNCs (at day 7). White boxed areas in the upper images (scale bar, 100 µm) were magnified in the lower images (scale bar, 100 µm). **(B)** Flow cytometric analysis of FSC/SSC gated cells, CD11b^+^/CD206^−^ (Mono-naïve) and CD11b^+^/CD206^+^ (M2) macrophages, among PBMNCs (at day 0) and E-MNCs (at day 7). **(C)** Percentages of Mono-naïve (CD11b^+^/CD206^−^) and M2 (CD11b^+^/CD206^+^) macrophage fractions and their ratios (Mono-naïve/M2) among PBMNCs and E-MNCs (*p < 0.05). **(D)** Flow cytometric analysis of CD11b^+^/Msr1^+^ (M2c; left panels) macrophages and CCR2/galectin3^+^ (right panels) CD11b-positive macrophages among PBMNCs (at day 0) and E-MNCs (at day 7). **(E)** Flow cytometric analysis of CCR6^−^/CCR4^+^ Th2 cells in CD3^+^/CD4^+^ gated cells of PBMNCs (at day 0) and E-MNCs (at day 7). **(F)** Expression of pro-inflammatory mRNAs (*il-1β*, *ifn-γ*, and *tnf-α*) in PBMNCs (at day 0) and E-MNCs (at day 7) (*p < 0.05, **p < 0.01). For statistical analysis, the Student’s t-test was performed to determine the significance of differences among PBMNCs and E-MNCs.

Among E-MNCs obtained from CB6F1 mice, adherent cells also became larger and changed their morphology to macrophage-like round or elongated shapes, similar to human E-MNCs (Figure 3A) at 7 days of culture. Flow cytometric analysis clearly revealed that the M2 macrophage-enriched fraction (CD11b^+^/CD206^+^) increased among E-MNCs (from 0.26% ± 0.22% to 5.54% ± 4.04%) (Figure 3B). The ratio of Mono-naïve macrophages (CD11b^+^/CD206^−^) among E-MNCs exhibited minimal variation (from 4.2% ± 1.89% to 5.56% ± 2.32%; no statistical differences between PBMNCs and E-MNCs), and the Mono-naïve/M2 macrophage ratio in E-MNCs decreased compared with that in PBMNCs (from 12.71 ± 3.42 to 1.35 ± 0.70) (Figure 3C). The fraction shifted to a predominance of immunomodulatory M2 macrophages (Msr1^+^/CD11b^+^; from 1.33% ± 1.07% to 2.97% ± 1.47%) (Figure 3D). Focusing particularly on the CD11b-positive cell fraction among PBMNCs and E-MNCs, galectin3^+^ cells (as immunomodulatory macrophages) clearly increased (from 3.04% ± 0.66% to 57.60% ± 20.8%) after 5G-culture (Figure 3D). Th2 cells (CD3^+^/CD4^+^/CXCR3^−^/CCR6^−^/CCR4^+^) increased (from 0.090% ± 0.036% to 4.20% ± 0.95%; Figure 3E), whereas Th1 cells (CD3^+^/CD4^+^/CXCR3^+^) slightly decreased (from 1.90% ± 0.33% to 0.69% ± 0.44%; data not shown). Expression of pro-inflammatory mRNAs (*il-1β*, *inf-γ*, and *tnf-α*) in E-MNCs was significantly downregulated compared with that in PBMNCs (Figure 3F). Further microarray analyses indicated that overall, pro-inflammatory, anti-angiogenesis, and pro-apoptosis genes were downregulated in E-MNCs, whereas the expression of anti-inflammatory, angiogenesis, anti-apoptosis, and apoptosis clearance–related genes was upregulated when compared with PBMNCs (Supplementary Figure S1A). In addition, as previously indicated in human/mouse EPC-CFA, two types of EPC colonies were morphologically detected: PEPC-CFUs and DEPC-CFUs. The total number of CFUs and the ratio of DEPC-CFUs derived from E-MNCs increased markedly compared with PBMNCs (Supplementary Figures S1B, C). These colonies were positive for ILB4-FITC (ILB4; binds to endothelial cells) and took up the AcLDL-DiI fluorescent dye (AcLDL labels endothelial cells) (Supplementary Figure S1D). These results indicated that the E-MNCs had acquired vasculogenic characteristics during 5G-culture. Furthermore, experiments to determine whether E-MNCs can be produced from NOD mice as an animal model of SS, macrophage-like round or elongated shapes were observed in addition to CB6F1 mouse E-MNCs at 7 days of 5G-culture (Supplementary Figure S2A). The M2 macrophage-enriched fraction (CD11b^+^/CD206^+^) increased among E-MNCs after 5G-culture (from 1.05% to 3.49%) (Supplementary Figure S2B). Additionally, galectin3-positive cells (as immunomodulatory macrophages) clearly increased (from 18.19% to 90.6%) after 5G-culture (Supplementary Figure S2C).

### 3.3 Anti-inflammatory effect of E-MNCs against inflamed PBMNCs

**FIGURE 4:**
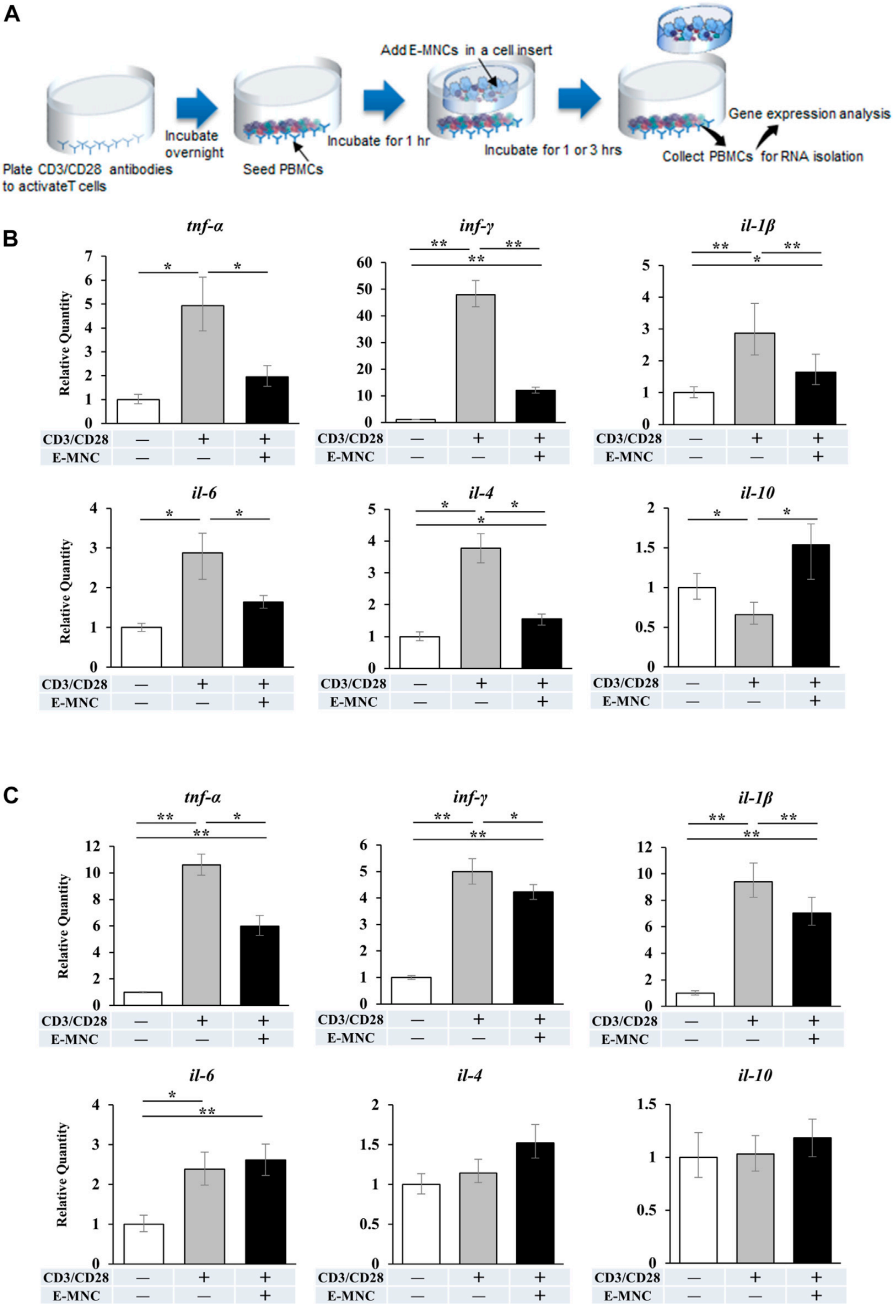
mRNA expression in CD3/CD28-stimulated PBMNCs after co-culture with E-MNCs. **(A)** Schematic diagram describing the experimental design for co-culture of E-MNCs and T-cell–activated PBMNCs. Anti-CD3 and -CD28 antibodies were added to the wells of a 24-well plate and incubated overnight. PBMNCs were then seeded in the wells and cultured at 37°C for 1 h. Subsequently, E-MNCs were seeded in the upper chamber and co-cultured with stimulated PBMNCs for 1 or 3 h **(B and C)** mRNA expression of *tnf-α*, *ifn-γ*, *il-1β*, *il-6*, *il-4*, and *il-10* in PBMNCs and CD3/CD28-stimulated PBMNCs with/without E-MNCs for 1 h **(B)** or 3 h **(C)** (*p < 0.05, **p < 0.01). For statistical analysis, one-way ANOVA with *post hoc* Tukey’s multiple comparisons were performed for multiple groups.

In preliminary experiments, mRNA expression of pro-inflammatory genes in CD3/CD28-stimulated PBMNCs peaked at around 2 h of incubation (data not shown). Therefore, E-MNCs were added to the culture at 1 h after stimulation of PBMNCs (Figure 4A). At 1 h after addition of E-MNCs, the E-MNCs downregulated the mRNA expression of *tnf-α*, *ifn-γ*, *il-1β*, *il-6*, and *il-4* in stimulated PBMNCs to the same levels as unstimulated PBMNCs, whereas E-MNCs upregulated the expression of anti-inflammatory *il-10* mRNA (Figure 4B). At 3 h, although E-MNCs significantly reduced mRNA expression of *tnf-α*, *inf-γ*, and *il-1β*, the anti-inflammatory effect of the E-MNCs decreased over time. Indeed, no significant change was observed in *il-6*, *il-4*, or *il-10* mRNA expression among stimulated PBMNCs co-cultured with/without E-MNCs (Figure 4C).

### 3.4 Therapeutic effects of E-MNCs on the onset of SS in NOD mice

**FIGURE 5:**
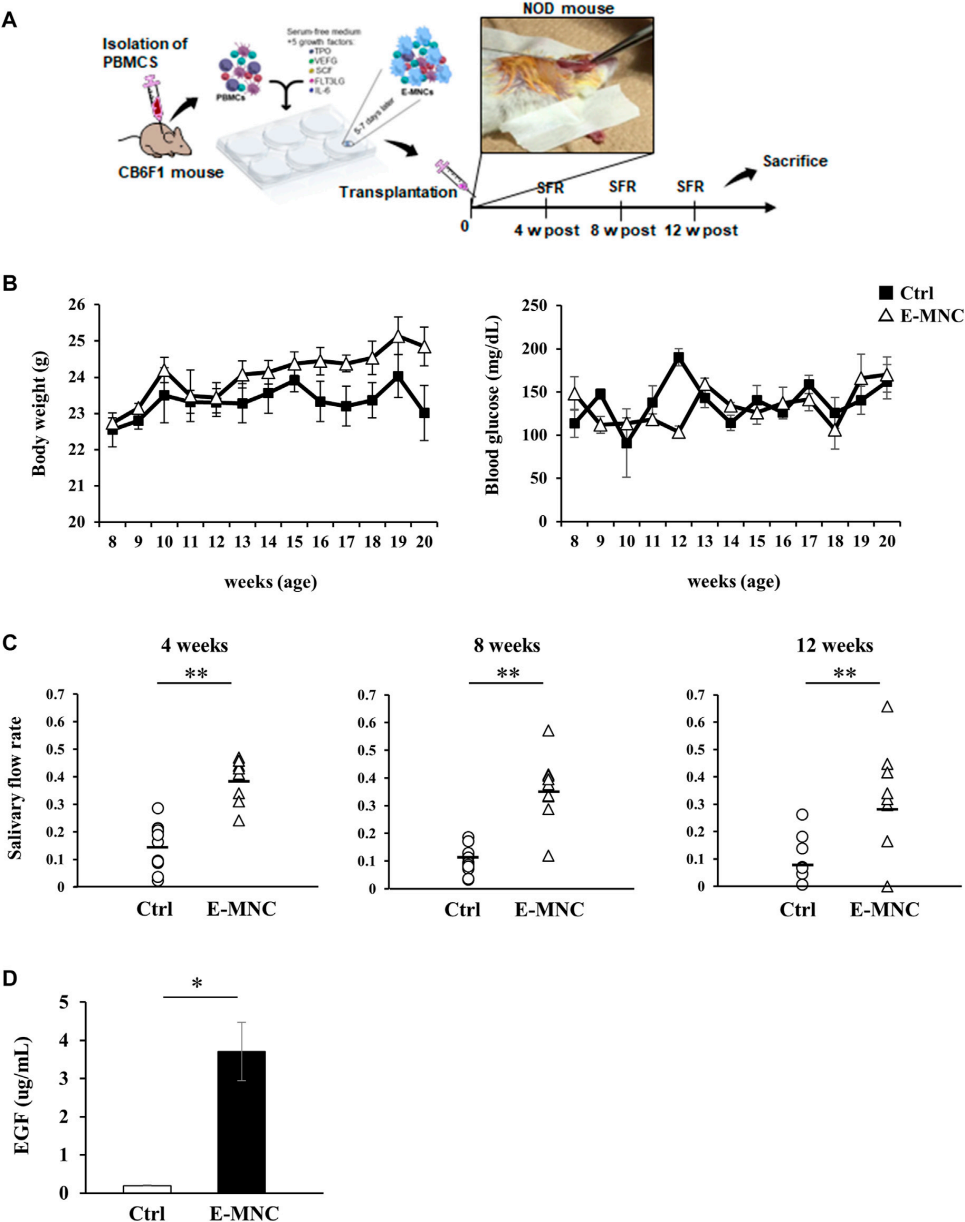
Transplantation of E-MNCs into NOD mice at the onset of SS-like disease. **(A)** Schematic diagram describing the experimental design for E-MNC transplantation. E-MNCs were injected into the submandibular glands directly, and then saliva and saliva glands were harvested at 4, 8, and 12 weeks. **(B)** Changes in body weight and blood glucose of non-treated mice (Ctrl) and E-MNC–treated mice (E-MNC). **(C)** Change in saliva production (salivary flow rate; SFR) at 4, 8, and 12 weeks post-transplantation (**p < 0.01). **(D)** EGF concentration in saliva of non-treated mice (Ctrl) and E-MNC–treated mice (E-MNC) at 12 weeks post-transplantation (*p < 0.05). For statistical analysis, the Student’s t-test was performed to determine the significance of differences among Ctrl- and E-MNC-group.

**FIGURE 6:**
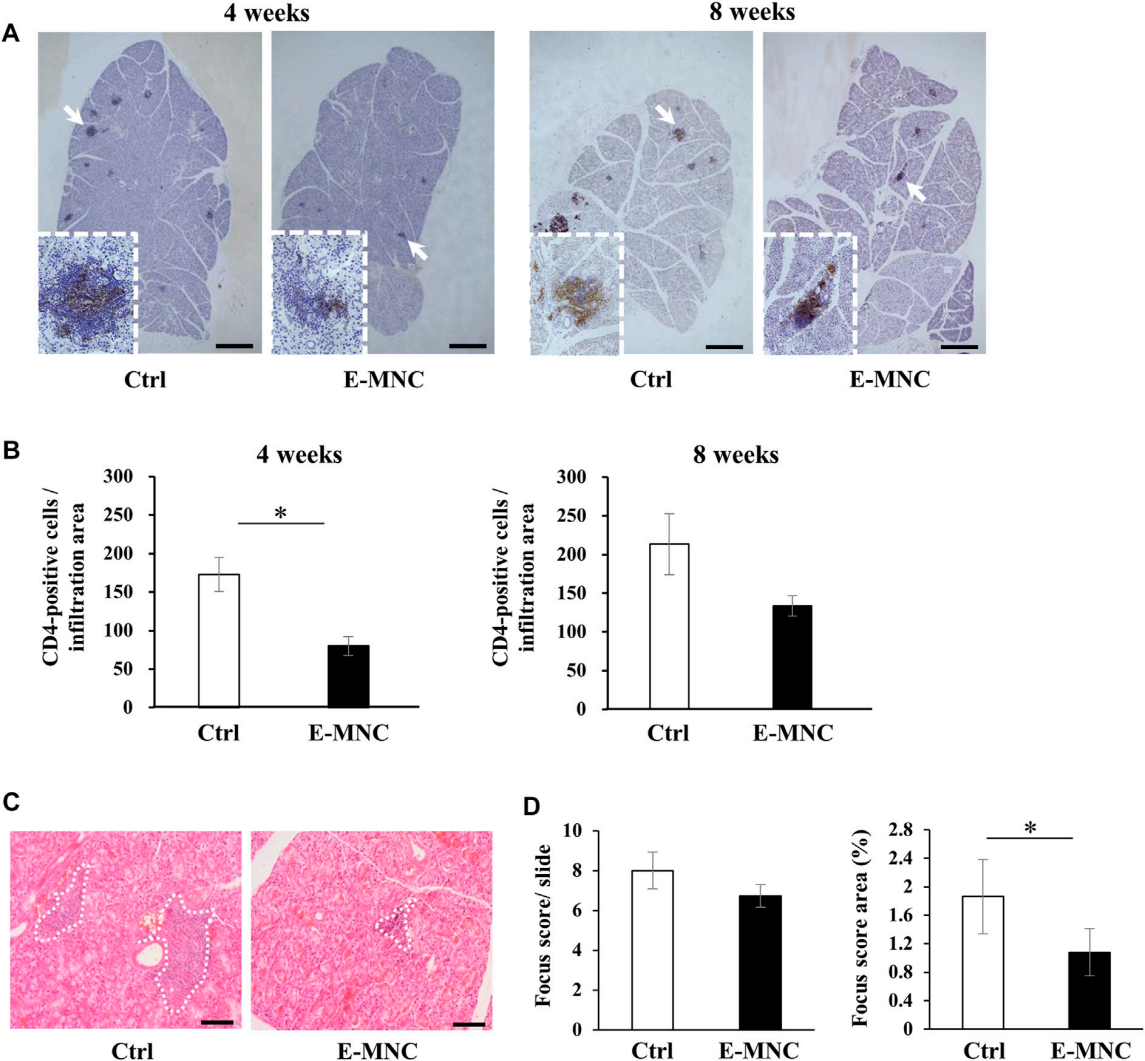
Lymphocyte infiltration after E-MNC treatment. **(A)** CD4 staining of submandibular glands in non-treated mice (Ctrl) and E-MNC–treated mice (E-MNC) at 4 and 8 weeks post-transplantation (scale bar, 1,000 μm). Cells positive for CD4 (T-helper cells) appear brown in the foci. **(B)** Number of CD4-positive cells in lymphocyte infiltrated areas at 4 and 8 weeks (*p < 0.05). **(C)** H&E staining of submandibular glands in non-treated mice (Ctrl) and E-MNC–treated mice (E-MNC) at 4 and 8 weeks post-transplantation (scale bar, 200 µm). White dotted lines indicate the portion used to determine the focus score. **(D)** Focus score and area in non-treated mice (Ctrl) and E-MNC–treated mice (E-MNC) (*p < 0.05). For statistical analysis, the Student’s t-test was performed to determine the significance of differences among Ctrl- and E-MNC-group.

E-MNCs obtained from PBMNCs of CB6F1 mice via 5G-culture were transplanted into the submandibular glands of 8-week-old NOD mice (Figure 5A). During the monitoring period up to 12 weeks post-transplantation, the body weight gradually increased after E-MNC treatment (Figure 5B). In contrast, the blood glucose level showed no clear difference between E-MNC–treated and non-treated mice (Figure 5B). However, 8- to 11-week-old NOD mice were considered to have developed SS, as lymphocyte infiltration began to be observed in SGs at these ages (data not shown). With regard to functional evaluation, E-MNC treatment clearly increased saliva secretion at 4, 8, and 12 weeks post-transplantation compared with non-transplanted mice (Figure 5C). The EGF concentration in harvested saliva at 12 weeks post-transplantation was markedly elevated in samples from E-MNC–treated mice compared to that in non-transplanted mice (Figure 5D). Areas of lymphocyte infiltration in submandibular glands appeared to decrease following E-MNC treatment at 4 and 8 weeks post-transplantation (Figure 6A). Indeed, the number of CD4-positive cells in lymphocyte infiltrated areas decreased markedly at 4 weeks in E-MNC–treated specimens compared with non-treated specimens (Figure 6B). However, after 8 weeks, the number of CD4-positive cells increased in both groups, though their numbers were still suppressed by E-MNC treatment (Figure 6B). At 12 weeks post-transplantation, the number of lymphocyte-infiltrated areas (focus score) in glands decreased slightly in E-MNC–treated specimens, whereas the average size of the lymphocyte-infiltrated areas was significantly reduced by E-MNC treatment (Figures 6C, D).

### 3.5 Behavior of transplanted E-MNCs in submandibular glands

**FIGURE 7:**
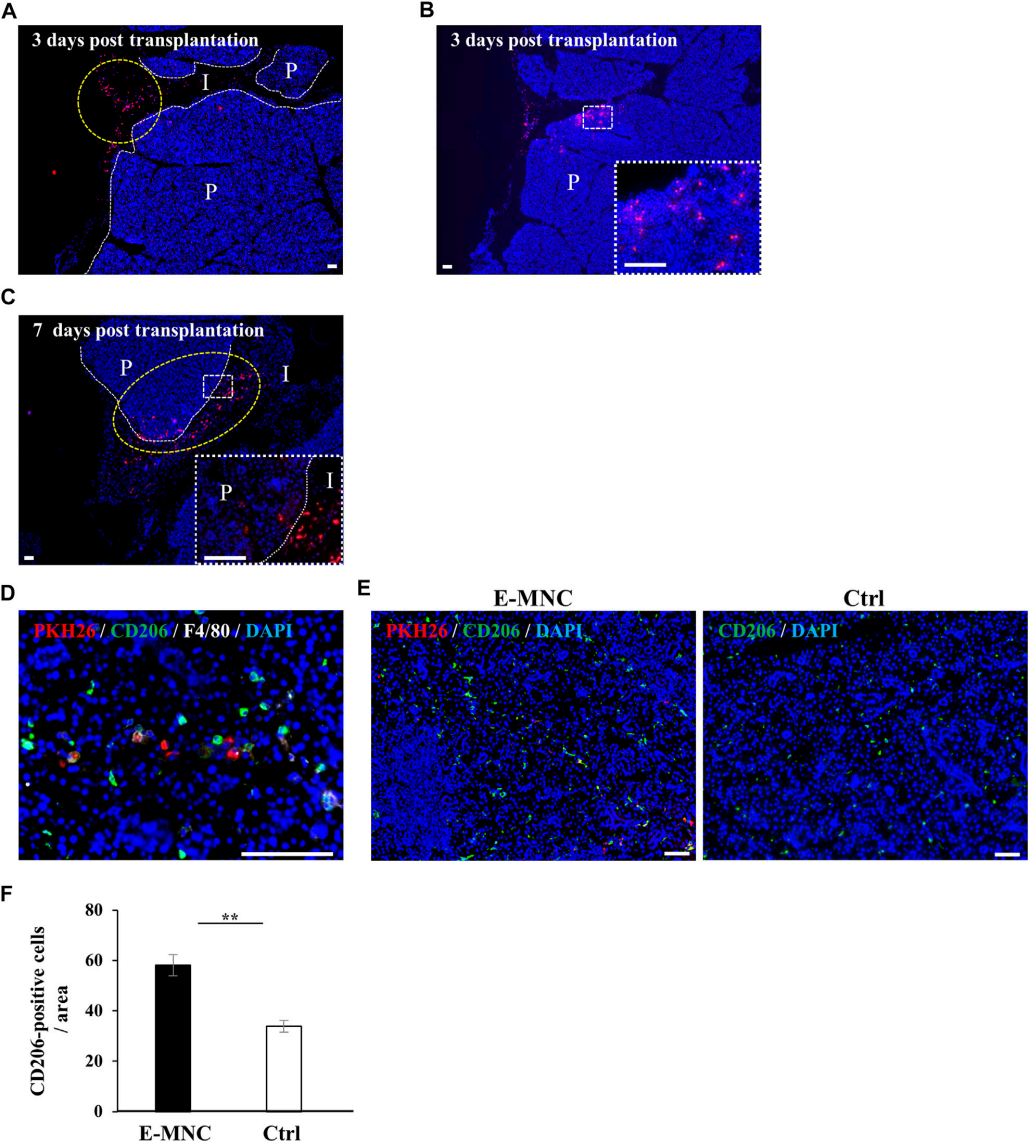
Detection of transplanted E-MNCs and M2 macrophages in submandibular glands. **(A)** At 3 days post-transplantation, PKH26-expressing E-MNCs (red) were detected in the interlobular space (I) (yellow dotted circle) and parenchyma (P) of submandibular glands (white dotted lines, boundary between interlobular area and parenchyma; red, PKH26-labeled cells; blue, DAPI; scale bar, 100 μm). **(B)** At 3 days post-transplantation, PKH26-expressing E-MNCs (red) were detected in the parenchyma (P) of submandibular glands. White dotted square area was magnified in the lower right panel (red, PKH26-labeled cells; blue, DAPI; scale bar, 100 μm). **(C)** At 7 days post-transplantation, PKH26-expressing E-MNCs (red) migrated to the deeper interlobular space (I) and penetrated into the parenchyma (P) (yellow dotted circle) in submandibular glands. White dotted square area was magnified in the lower right panel (white dotted lines, boundary between interlobular and parenchyma; red, PKH26-labeled cells; blue, DAPI; scale bar, 100 μm). **(D)** At 1 day post-transplantation, PKH26-expressing E-MNCs and host M2 macrophages expressed F4/80 (white) and CD206 (green) in the parenchyma of the submandibular glands (blue, DAPI; scale bar, 100 μm). **(E)** Detection of transplanted E-MNCs (red) and CD206-positive cells (green) in E-MNC-treated glands (left image) and non-treated glands (right image) (blue, DAPI; scale bar, 50 μm). **(F)** Number of CD206-positive cells/field (×200) in the parenchyma of submandibular gland tissues in E-MNC-treated glands (E-MNC) and non-treated glands (Ctrl) at 7 days post-transplantation (**p < 0.01). For statistical analysis, the Student’s t-test was used to analyze differences between E-MNC-group and Ctrl-group.

To assess the behavior of transplanted E-MNCs in submandibular glands, PKH26-labeled E-MNCs were analyzed on days 1, 3, and 7 after transplantation. At 3 days, scattered PKH26-labeled cells were primarily observed in the interlobular space (Figure 7A), from which some cells appeared to invade the gland parenchyma (Figures 7A, B). Subsequently, labeled cells migrated to the deeper space between the lobules (Figure 7C), and numerous cells penetrated the gland parenchyma at 7 days post-transplantation (Figure 7C). Observation of E-MNCs in the gland parenchyma at 1 day post-transplantation revealed F4/80/CD206-positive E-MNCs and host cells (as M2 macrophages) (Figure 7D). At 7 days, E-MNC–treated glands exhibited a higher number of cells positive for F4/80/CD206 (as M2 macrophages) than did non-treated glands (Figures 7E, F). In particular, a number of CD206-expressing host cells were seen at the periphery of PKH26-labeled E-MNCs containing CD206-positive cells (Figure 7E).

### 3.6 Gene expression in submandibular glands in the initial post-transplantation stage

**FIGURE 8:**
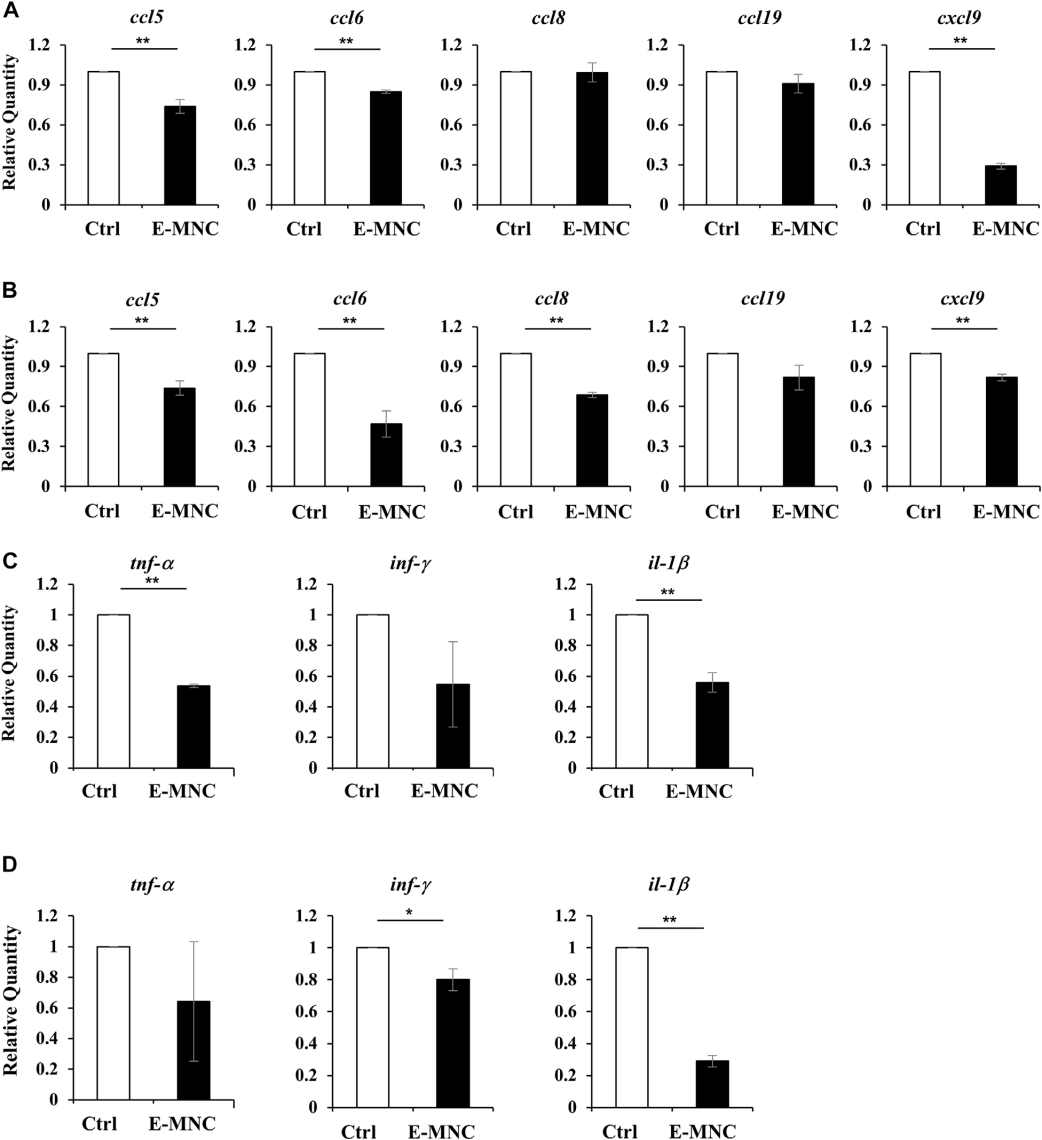
Gene expression in submandibular glands in the initial post-transplantation stage. **(A,B)** mRNA expression of the inflammatory chemokines *ccl5*, *ccl6*, *ccl8*, *ccl19*, and *cxcl9* in non-treated glands (Ctrl) and E-MNC–treated glands (E-MNC) at 1 day **(A)** and 3 days **(B)** post-transplantation (**p < 0.01). **(C,D)** Expression of the pro-inflammatory genes *tnf-α*, *inf-γ*, and *il-1β* in non-treated glands (Ctrl) and E-MNC–treated glands (E-MNC) at 1 day **(C)** and 3 days **(D)** post-transplantation (*p < 0.05, **p < 0.01). For statistical analysis, the Student’s t-test was used to analyze differences between E-MNC-group and Ctrl-group.

To further assess the therapeutic effects underlying the anti-inflammatory activity of E-MNCs, the expression of inflammatory chemokine genes, especially genes related to lymphocyte infiltration, was examined in the initial post-transplantation stage. At 1 day post-transplantation, *ccl5*, *ccl6*, and *cxcl9* mRNA expression was significantly downregulated in E-MNC–treated glands, but no apparent decline in expression of the *ccl8* and *ccl19* genes was observed (Figure 8A). Subsequently, mRNA expression of these chemokines in E-MNC–treated glands was generally suppressed at 3 days compared with that in non-treated glands (Figure 8B). Consistent with these observations, the expression of pro-inflammatory genes such as *tnf-α*, *inf-γ*, and *il-1β* was markedly downregulated at both 1 and 3 days post-transplantation (Figures 8C, D).

## 4 Discussion

This study demonstrated that a cell therapy approach based on E-MNCs has a therapeutic efficacy on the onset of pSS-like disease in NOD mice. The major findings of this study were as follows: 1) transplanted E-MNCs obtained from peripheral blood consistently preserved salivary secretion, 2) E-MNCs clearly suppressed the infiltration of CD4-positive lymphocytes into SGs and the progression of SS-like disease, and 3) E-MNCs might affect these phenomena in a paracrine manner and/or in cooperation with induced recipient anti-inflammatory M2 macrophages. These outcomes suggest that this strategy could be a promising option for developing future treatments.

Regarding the first outcome, NOD mice exhibited lower levels of saliva output over time, but the levels were maintained near those of normal mice in E-MNC–treated mice when compared with NOD mice (2.46-fold at 12 weeks; 3.59-fold at 16 weeks; 3.19-fold at 20 weeks of age). This therapeutic effect on SG function was preserved during the 12-week observation period following a single administration of E-MNCs. These phenomena indicate that E-MNCs exert a certain therapeutic efficacy in preventing the progression of SS-like disease or restoring the secretory function of injured glands. In previous studies, we demonstrated that administration of bone marrow MSCs is effective for both preventing lymphocytic infiltration and suppressing hyposalivation in SGs of NOD mice (Khalili et al., 2012; Khalili et al., 2014; Abughanam et al., 2019). Also, other studies have reported similar therapeutic effects of MSCs in treating NOD mice with SS-like disease (Shi et al., 2018; Gong et al., 2020; Cong et al., 2022; Sun et al., 2022). The anti-inflammatory and immunomodulatory properties of MSCs are responsible for the therapeutic effects against SS-like disease. Therefore, MSCs are ideal candidates for cell-based therapies. However, as mentioned above, the availability of autologous MSCs for effective therapy may be limited due to the relatively low number of cells that can be obtained from donor tissues, degeneration of their plasticity during cell expansion and passaging, patient age, and invasiveness. In contrast, E-MNCs exhibit promise for clinical applications because the peripheral blood is a readily accessible source of cells that can be obtained with minimal invasiveness in elderly patients. The most significant advantage is that this functional primary culture system can induce immunomodulatory cells into E-MNCs from a patient’s peripheral blood. Furthermore, E-MNCs may contribute to the functional restoration of atrophic glands more directly than MSCs, because exogenous MSCs play a role in tissue regeneration by inducing macrophage polarization toward an anti-inflammatory M2 phenotype (Nemeth et al., 2009; Zhang et al., 2010). The human E-MNCs in this study were approximately 24.5% M2-dominant monocytes/macrophages (0.5% CD11b^+^/CD206^−^ Mono-naïve macrophages; 24% CD11b^+^/CD206^+^ M2 macrophages). We found that this heterogeneous cell fraction exhibited anti-inflammatory effects against activated PBMNCs. The M2 macrophage fraction was also enriched among CB6F1 mouse E-MNCs, although their abundance was approximately 4%. However, genes associated with Th1 cells, such as *tnf-α*, *inf-γ*, and *il-1β*, were significantly downregulated, while the fraction of Th2 cells associated with M2 macrophage polarization increased during 5G-culture. In addition, a notable number of Msr1-or galectin3-expressing immunomodulatory macrophages were observed among E-MNCs. Overall, these data indicate that mouse E-MNCs consistently exhibit anti-inflammatory and immunomodulatory properties. Additionally, the results of flow cytometric analyses showed that E-MNCs obtained from NOD mice at the onset of pSS acquired an anti-inflammatory and immunoregulatory profile following 5G-culture, similar to those of CB6F1 mice. These results suggest that this culture system could overcome the inherent limitations of autologous cell therapy in patients with autoimmune diseases such as SS and that E-MNCs ultimately generated using this culture system may be useful as a therapeutic agent. Thus, the use of E-MNCs, which does not require long-term cell expansion, is a simple and direct approach with low invasiveness that takes advantage of a readily available source (blood).

With regard to inhibition of disease development, we found that E-MNC treatment inhibited the infiltration of CD4^+^ T cells at 4 and 8 weeks post-transplantation and reduced the area of lymphocyte infiltration (focus score area) at 12 weeks to approximately 58% of that of non-treated SGs. These phenomena indicate that transplanted E-MNCs directly suppress the infiltration of inflammatory cells and were not involved in the pathogenesis of pSS. As mentioned above, E-MNCs were enriched in M2 macrophages and Th2 cells. Typical M2 macrophages have the capacity to potently suppress CD4^+^ T cell proliferation via the release of IL-10 (Salminen, 2010). Interestingly, a histological study of lip SGs from pSS patients showed that the number of CXCR3^+^/CD163^+^ M2 macrophages was inversely correlated with the severity of inflammatory lesions, demonstrating that M2 macrophages play an anti-inflammatory role in pSS lesions (Aota et al., 2018; Ushio et al., 2018). Similarly, this study revealed that the proliferation of CD206^+^ M2 macrophage after E-MNC treatment was followed by suppression of CD4^+^ T cell proliferation in the submandibular glands. Considered collectively, these data suggest that among E-MNCs, M2 macrophages and/or supporting cells such as Th2 cells contribute to the prevention of lymphocyte infiltration in the initial stage of pSS.

**FIGURE 9:**
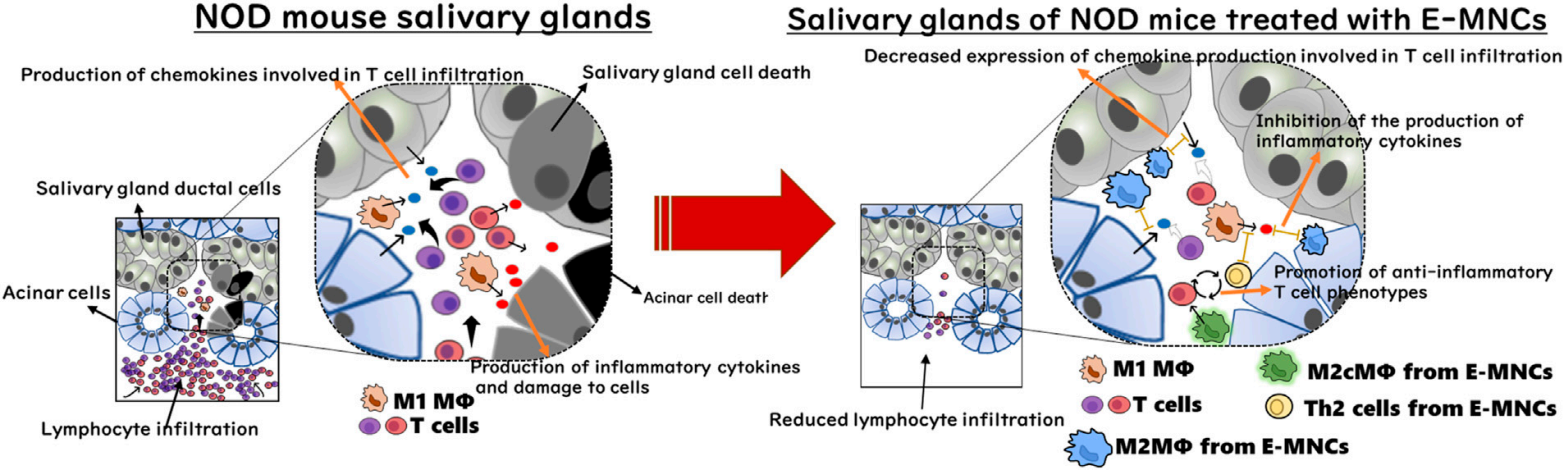
Diagram of the cellular mechanism of E-MNC transplantation at the onset of SS in NOD mice. E-MNCs function in a paracrine manner to inhibit chemotaxis of pathogenic T cells and improve the tissue microenvironment via the immunomodulatory effects of M2 macrophages and/or Th2 cells among E-MNCs during the initial stage after transplantation.

With regard to the cellular function of E-MNCs, human E-MNCs have been shown to potently suppress the activation of T cells among PBMNCs in culture, and mouse E-MNCs exhibit downregulation of mRNA expression of chemokines associated with T cell chemotaxis after transplantation (Huang et al., 2019; Chen et al., 2022). These data suggest that E-MNCs affect pathogenic T cells in a paracrine manner and improve the local inflammatory environment in injured SGs. In contrast, we found that transplanted E-MNCs gradually migrate from the interlobular space to the parenchyma over time. However, interestingly, CD206^+^ M2 macrophages were derived from not only E-MNCs but also from the host cells could be observed in treated glands from 1day post-transplantation. As mentioned above, CCR4^+^/CCR6^−^ Th2 cells were also enriched in E-MNCs. Th2 cells are known to produce anti-inflammatory cytokines such as IL-4, IL-10, IL-13, and TGF-β (Young et al., 2000; Wegmann, 2009; Iwaszko et al., 2021; Kokubo et al., 2022). Moreover, galectin3 produced by macrophages plays a major role in the activation of M2 polarization via IL-4 signaling in Th2 cells (MacKinnon et al., 2008; Sperandio et al., 2009; Espinosa Gonzalez et al., 2022). Therefore, M2 macrophages and Th2 cells among E-MNCs might function in cooperation with self-induced host M2 macrophages. These findings led us to conclude that E-MNCs act in a paracrine manner to inhibit the chemotaxis of pathogenic T cells and improve the tissue microenvironment via immunomodulatory functions of M2 macrophages and/or Th2 cells among E-MNCs during the initial post-transplantation stages (Figure 9). However, the data in this study are still limited due to the lack of quantitative analysis at the protein level of chemokines such as CCL5, CCL6, CCL8, CCL19, and CXCL9, which are remarkably induced by pathogenic T cells at the disease initiation. During the development of SS-like disease, multiple chemokines function in a coordinated manner, with these chemokine-ligand families exhibiting different phases of gene expression (Nguyen CQ et al., 2009). Therefore, there may be no apparent differences in protein level of each chemokine because the changes in their mRNA expressions are relatively small (within one to three times). Overall changes in each chemokine production must be involved in the migration of pathogenic T cells. To resolve such limitations, further investigation on dynamic expression analysis of chemokine, chemokine receptor and cytokine gene responses in the pathological condition after E-MNC treatment may be required.

Msr1, an important phagocytic receptor in M2 macrophages, is involved in the elimination of apoptotic cells (Geske et al., 2002; Todt et al., 2008; Zhang et al., 2019; Zheng et al., 2022). Therefore, Msr1-expressing M2 macrophages among E-MNCs might contribute to alleviation of the inflammatory microenvironment and the activation of tissue regeneration. Indeed, the EGF concentration in saliva significantly increased after E-MNC treatment. EGF promotes the growth of salivary epithelial cells and inhibits apoptotic cell death (Azuma et al., 2018; Cho et al., 2021). It has been shown that the concentration of EGF increases in SGs of NOD mice with pSS-like disease following administration of MSCs (Khalili et al., 2012). Furthermore, as we and other groups demonstrated in a previous study (Masuda et al., 2011; Tsukada et al., 2013; I et al., 2019) using a CFU assay and immunocytochemistry (uptake of AcLDL and binding of ILB), E-MNCs function both directly and indirectly in promoting angiogenesis during tissue regeneration. Data from microarray analyses also suggested that E-MNCs are involved in anti-inflammatory processes, angiogenesis, and the clearance of apoptotic cells. Some cells among E-MNCs must directly ameliorate the inflammatory microenvironment in injured SGs to facilitate tissue regeneration.

## 5 Conclusion

In conclusion, this study demonstrated that E-MNC therapy partially prevents the development of pSS-like disease in mice and preserves saliva secretion. E-MNCs can be produced using a readily available and minimally invasive source of cells in a short period of time. This therapy can be easily performed for SS patients in the clinic. However, the associated cellular mechanisms and therapeutic conditions need to be investigated in greater detail for future clinical applications. For example, this study did not focus on pathogenic B cells, which predominate in the chronic inflammatory stage of SS. Another limitation of the present study is that we employed a single transplantation to the submandibular glands using a specific dose, but tailored regimens that maximize the efficacy of E-MNC treatment should be developed. Therefore, further investigations employing proper clinical models of SS are needed. Overall, this study found that the immunomodulatory effects of E-MNCs could be part of a therapeutic strategy targeting the early stages of pSS.

## Data Availability

The original contributions presented in the study are included in the article/Supplementary Material, further inquiries can be directed to the corresponding author.
