# Supplementary material for: Effective-mononuclear cell (E-MNC) therapy alleviates salivary gland damage by suppressing lymphocyte infiltration in Sjögren-like disease
Source: Front Bioeng Biotechnol. 2023 Apr 24;11:1144624. doi: 10.3389/fbioe.2023.1144624 (PMC10164970; doi:10.3389/fbioe.2023.1144624)
Supplement: Supplementary file 1 [file DataSheet1.docx]

**Supplementary Material**

**Effective-mononuclear cell (E-MNC) therapy alleviates salivary gland damage by suppressing lymphocyte infiltration in Sjögren-like disease**

**Kayo Hasegawa^1†^, Jorge Luis Montenegro Raudales^1†^, Takashi I^1†^, Takako Yoshida^1^, Ryo Honma^1,2^, Mayumi Iwatake^1^, Simon D Tran^3^, Makoto Seki^4^, Izumi Asahina^2,5^ and Yoshinori Sumita^1*^**

^†^Kayo Hasegawa, Jorge Luis Montenegro Raudales, and Takashi I contributed equally to this work and share first authorship

***Correspondence:** Yoshinori Sumita**:** [y-sumita@nagasaki-u.ac.jp](mailto:y-sumita@nagasaki-u.ac.jp)


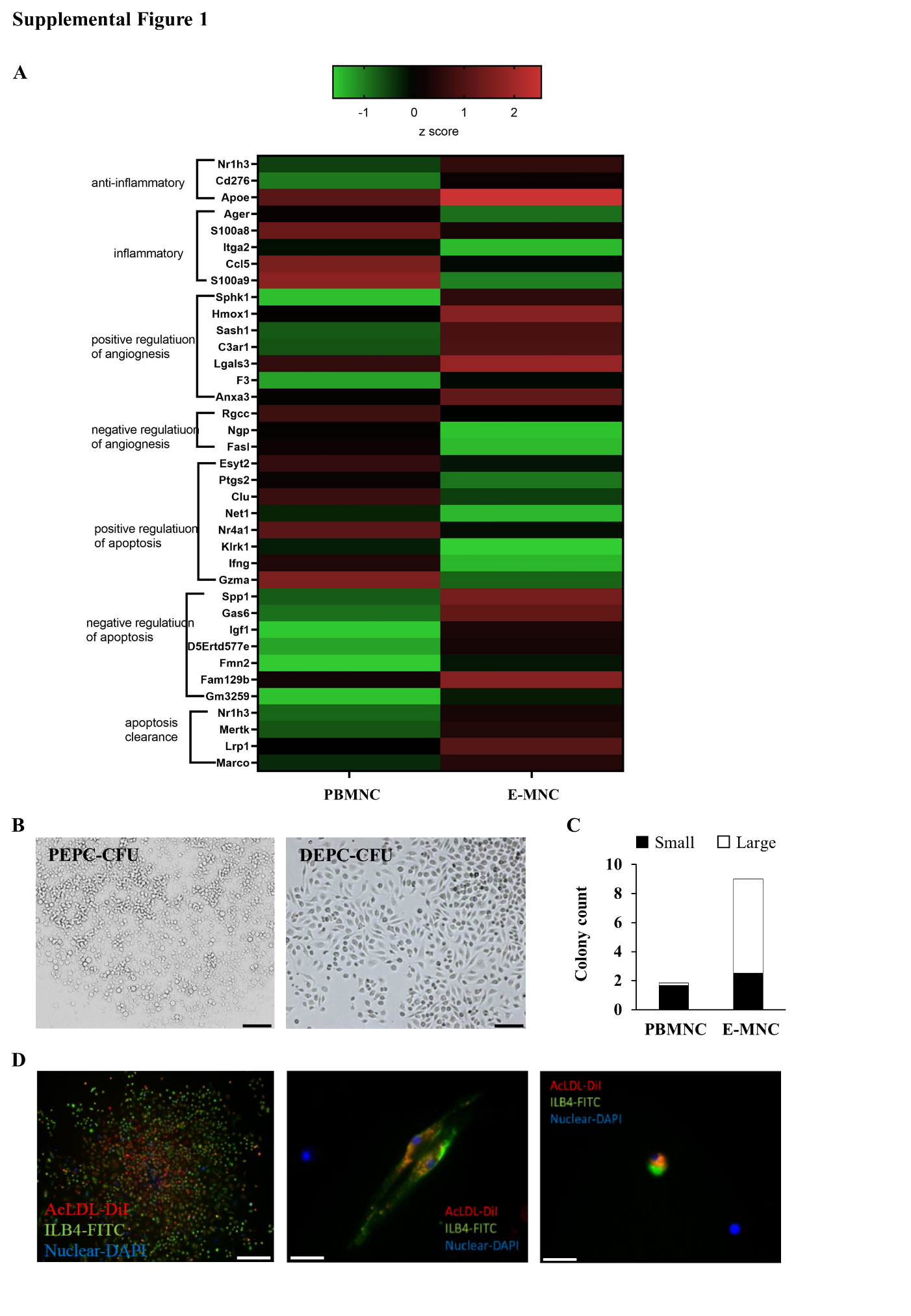


**Supplemental Figure 1.** Characteristics of mouse E-MNCs. **(A)** Microarray analysis of PBMNCs and E-MNCs. Heatmap showing down-regulated and up-regulated genes associated with anti-inflammation, inflammation, positive regulation of angiogenesis, negative regulation of angiogenesis, positive regulation of apoptosis, negative regulation of apoptosis, and apoptotic cell clearance in PBMNCs (day 0) and E-MNCs (day 7). Red color indicates relatively higher expression, and green color indicates relatively lower expression. **(B)** Representative images of EPC-CFUs at 7 days of EPC-CFA. Two types of EPC-CFU were observed: primitive EPC-CFUs (PEPC-CFUs) and definitive EPC-CFUs (DEPC-CFUs) (scale bar, 100 μm). **(C)** Number of EPC-CFUs from PBMNCs (PBMNC) and E-MNCs (E-MNC) per dish (1 × 10^5^ cells/dish). **(D)** Left image shows ILB4-conjugated FITC (green) binding and AcLDL-DiI (red) uptake of EPC-CFUs (blue, DAPI) (scale bar, 100 μm). Center and right images show large and small types of cells, composed of DEPC-CFUs and PEPC-CFUs, respectively (green, ILB4-FITC; red, AclDL-DiI; blue, DAPI) (scale bar, 50 μm).


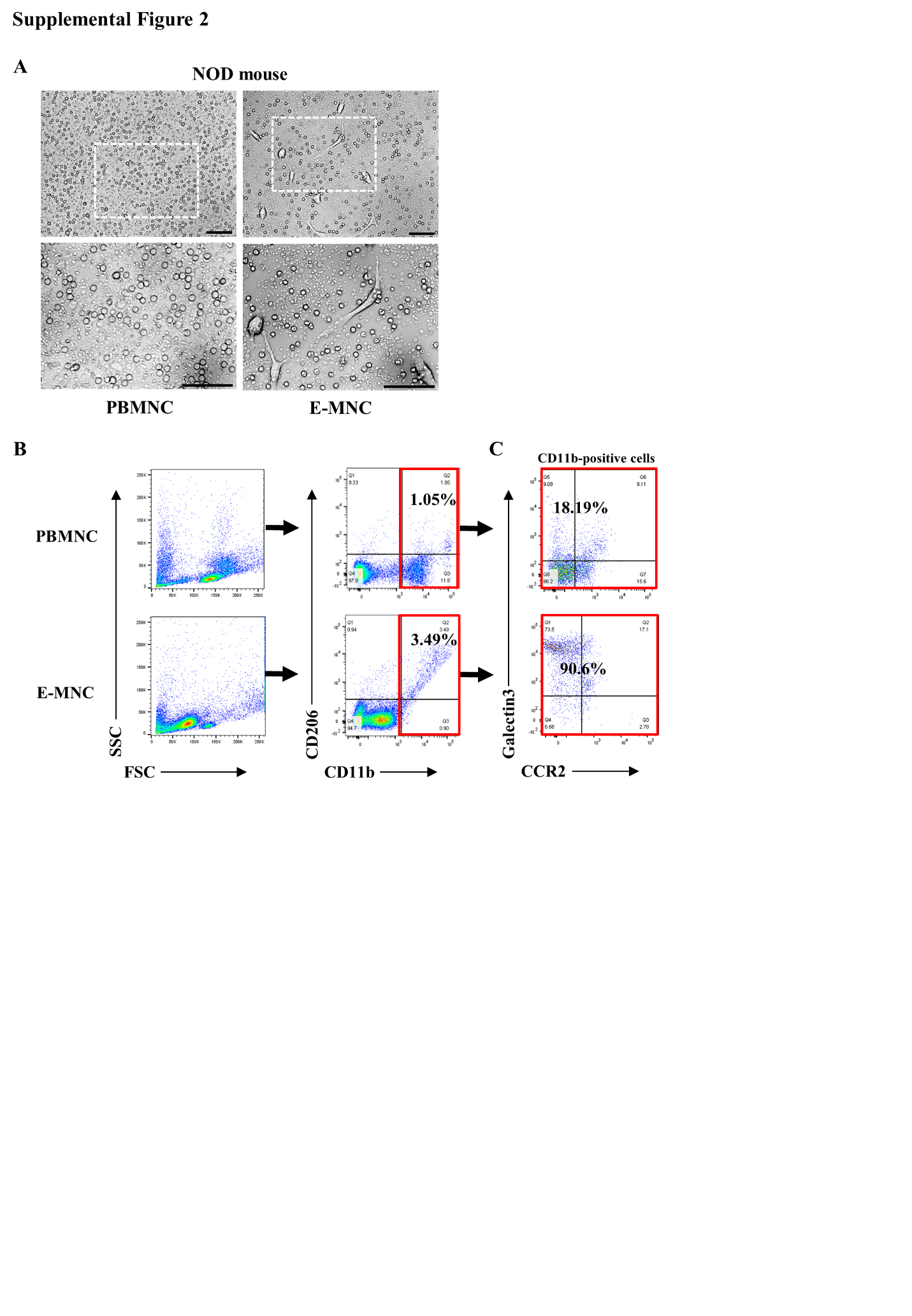


**Supplemental Figure 2.** Characteristics of NOD mouse E-MNCs. **(A)** Phase-contrast imaging of NOD mouse PBMNCs (at day 0) and E-MNCs (at day 7). White boxed areas in the upper images (scale bar, 100µm) were magnified in the lower images (scale bar, 100µm). **(B)** Flow cytometric analysis of FSC/SSC gated cells, CD11b^+^/CD206^−^ (Mono-naïve) and CD11b^+^/CD206^+^ (M2) macrophages, among PBMNCs (at day 0) and E-MNCs (at day 7). **(C)** Flow cytometric analysis of CCR2/galectin3^+^ CD11b-positive macrophages among PBMNCs (at day 0) and E-MNCs (at day 7).
